# Supplementary figures and images for: Over-expression of the photoperiod response regulator ZmCCT10 modifies plant architecture, flowering time and inflorescence morphology in maize
Source: PLoS One. 2019 Feb 6;14(2):e0203728. doi: 10.1371/journal.pone.0203728 (PMC6364868; doi:10.1371/journal.pone.0203728)

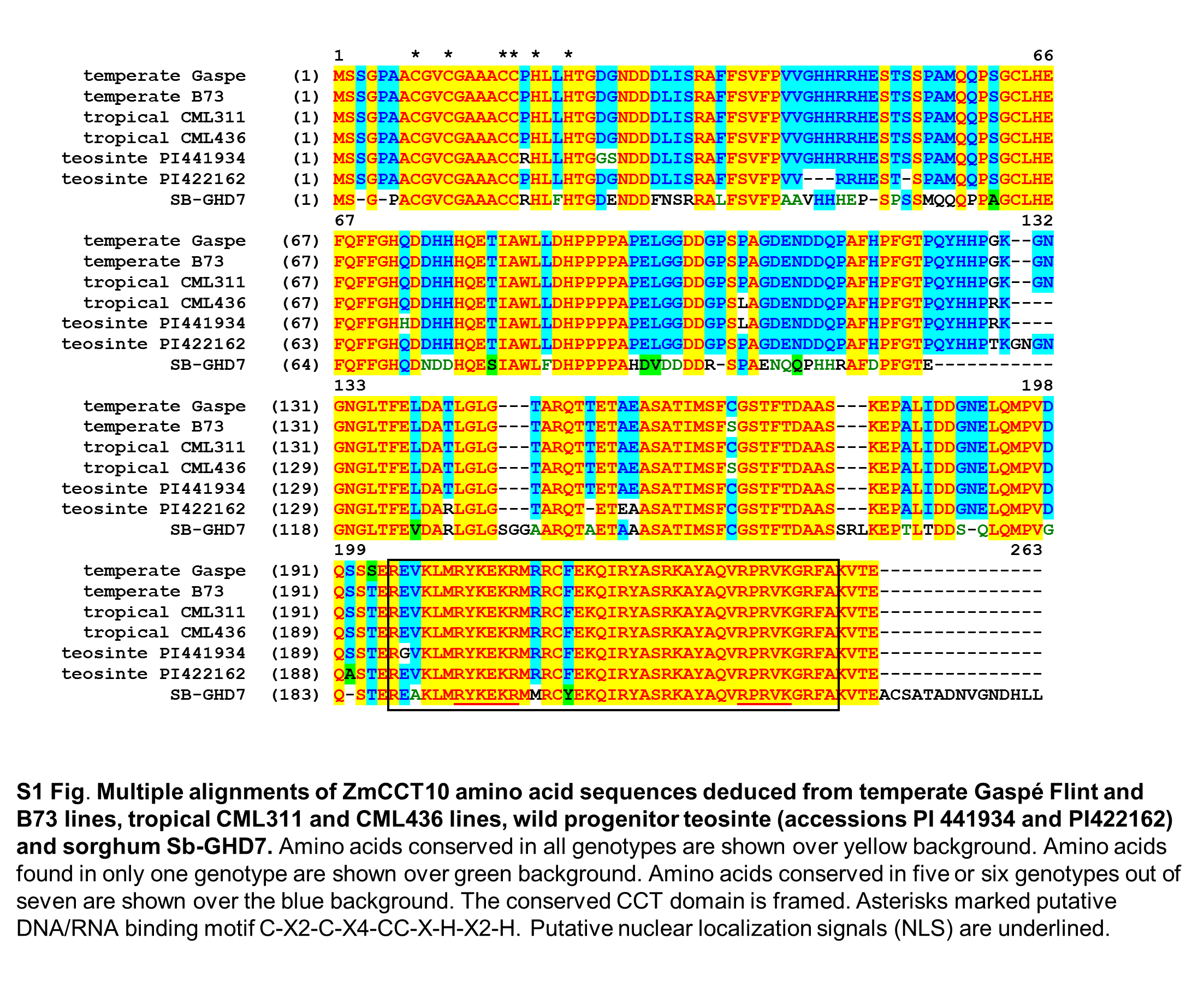

Supplement: S1 Fig — Amino acids conserved in all genotypes are shown over yellow background. Amino acids found in only one genotype are shown over green background. Amino acids conserved in five or six genotypes out of seven are shown over the blue background. The conserved CCT domain is framed. Asterisks marked putative DNA/RNA binding motif C-X2-C-X4-CC-X-H-X2-H. Putative nuclear localization signals (NLS) are underlined. (TIF) [file pone.0203728.s001.tif]

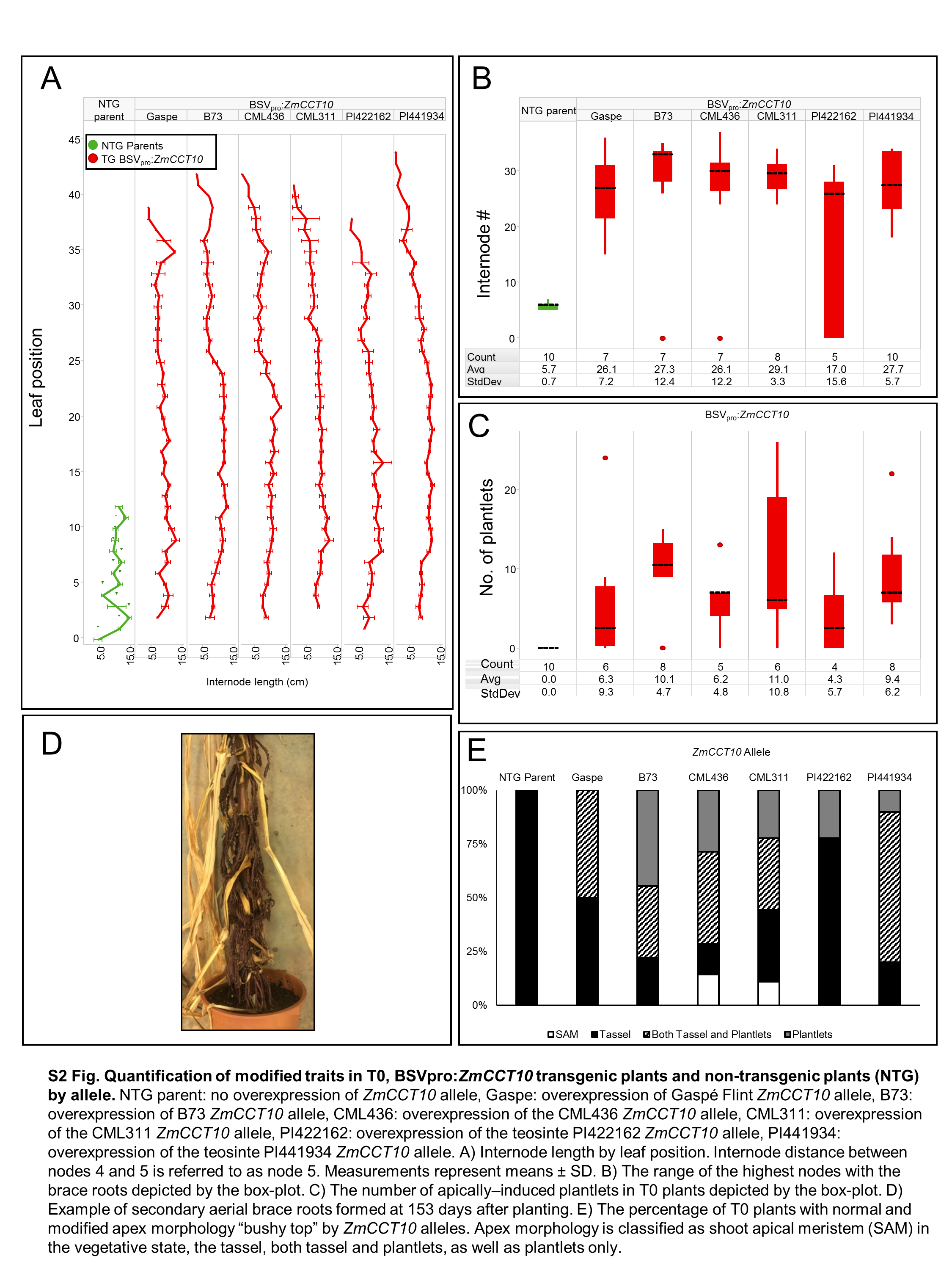

Supplement: S2 Fig — NTG parent: no overexpression of ZmCCT10 allele, Gaspe: overexpression of Gaspé Flint ZmCCT10 allele, B73: overexpression of B73 ZmCCT10 allele, CML436: overexpression of the CML436 ZmCCT10 allele, CML311: overexpression of the CML311 ZmCCT10 allele, PI422162: overexpression of the teosinte PI422162 ZmCCT10 allele, PI441934: overexpression of the teosinte PI441934 ZmCCT10 allele. A) Internode length by leaf position. Internode distance between nodes 4 and 5 is referred to as node 5. Measurements represent means ± SD. B) The range of the highest nodes with the brace roots depicted by the box-plot. C) The number of apically–induced plantlets in T0 plants depicted by the box-plot. D) Example of secondary aerial brace roots formed at 153 days after planting. E) The percentage of T0 plants with normal and modified apex morphology (phyllody)”top by ZmCCT10 alleles. Apex morphology is classified as shoot apical meristem (SAM) in the vegetative state, the tassel, both tassel and plantlets, as well as plantlets only. (TIF) [file pone.0203728.s002.tif]

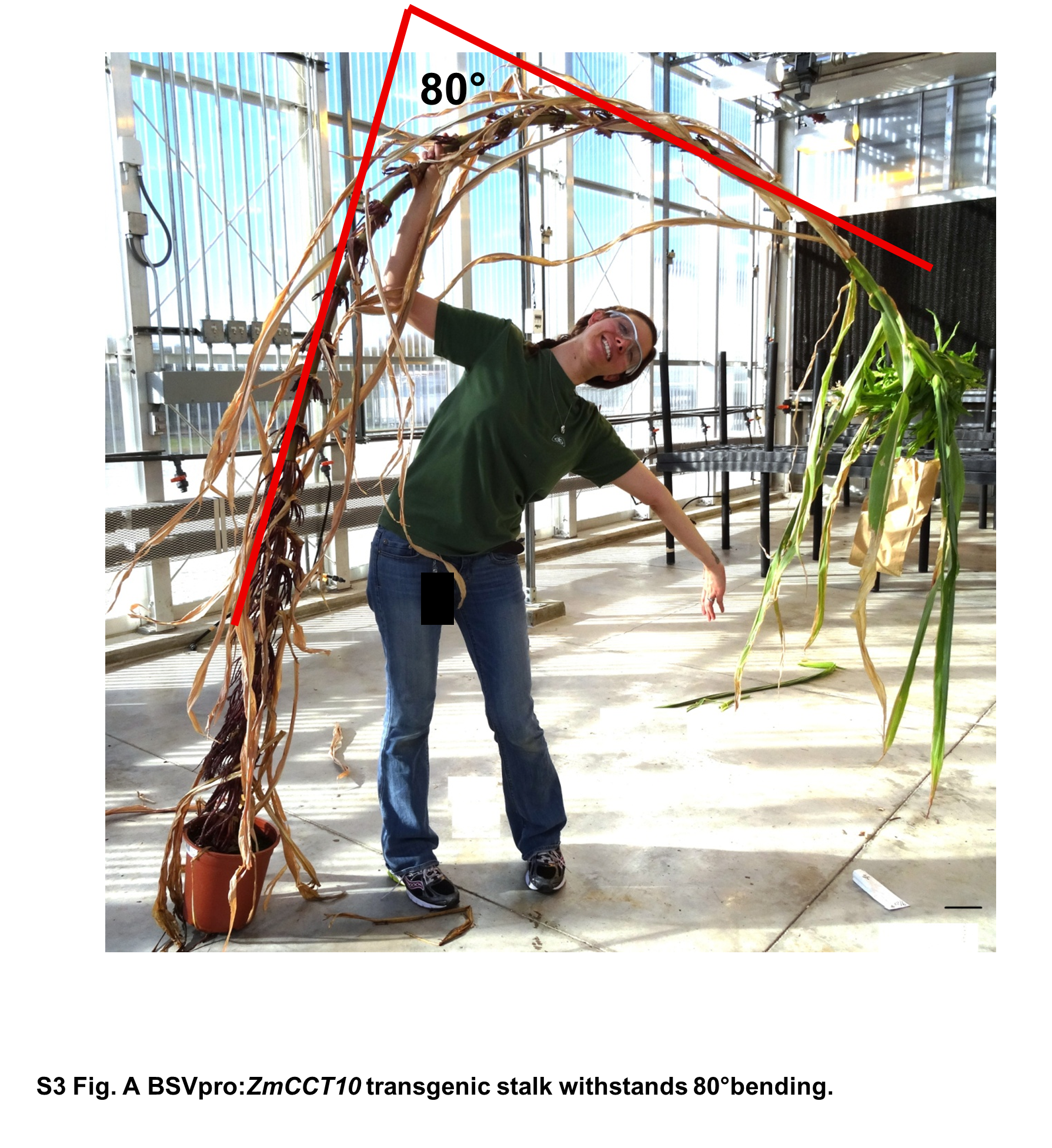

Supplement: S3 Fig — (TIF) [file pone.0203728.s003.tif]

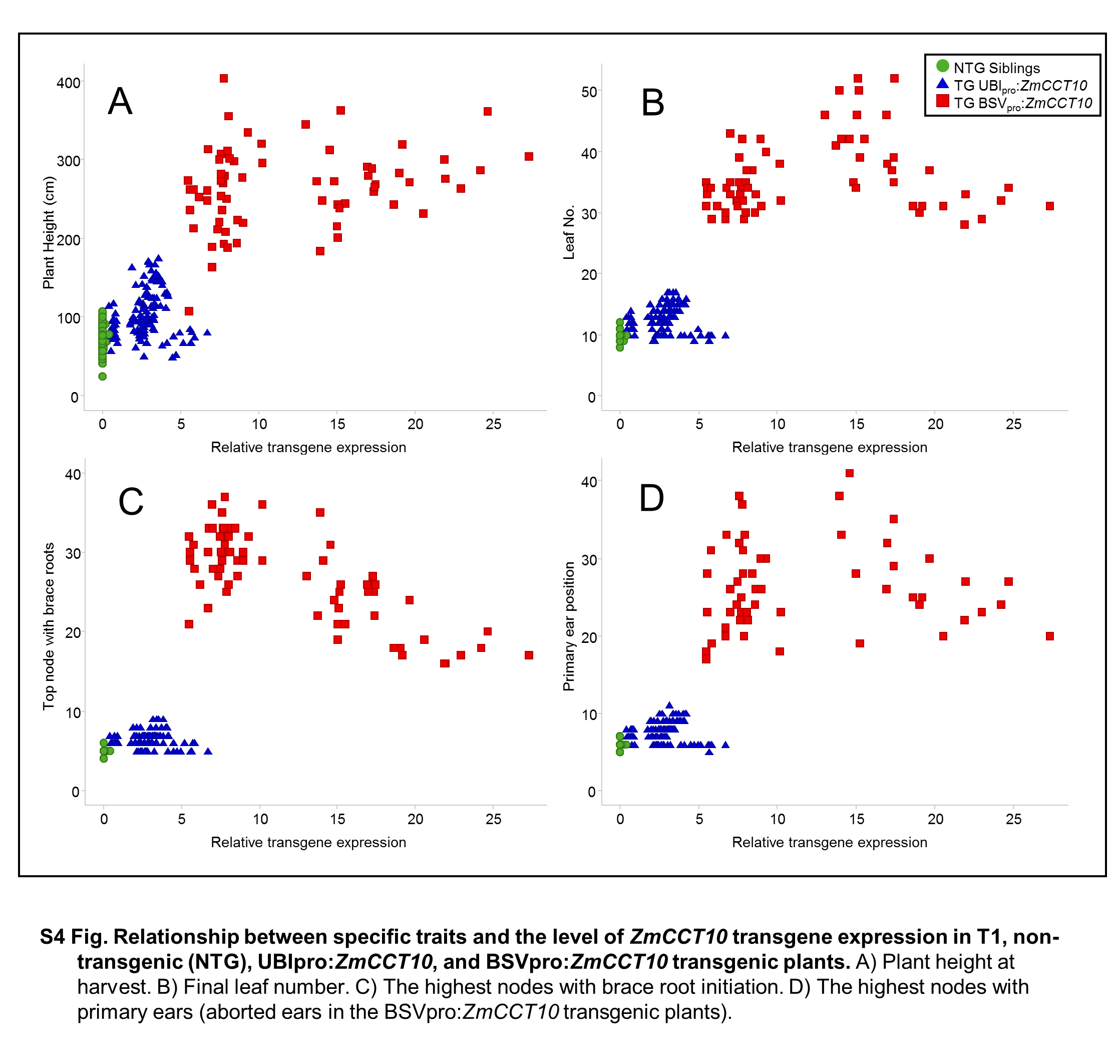

Supplement: S4 Fig — A) Plant height at harvest. B) Final leaf number. C) The highest nodes with brace root initiation. D) The highest nodes with primary ears (aborted ears in the BSVpro:ZmCCT10 transgenic plants). (TIF) [file pone.0203728.s004.tif]

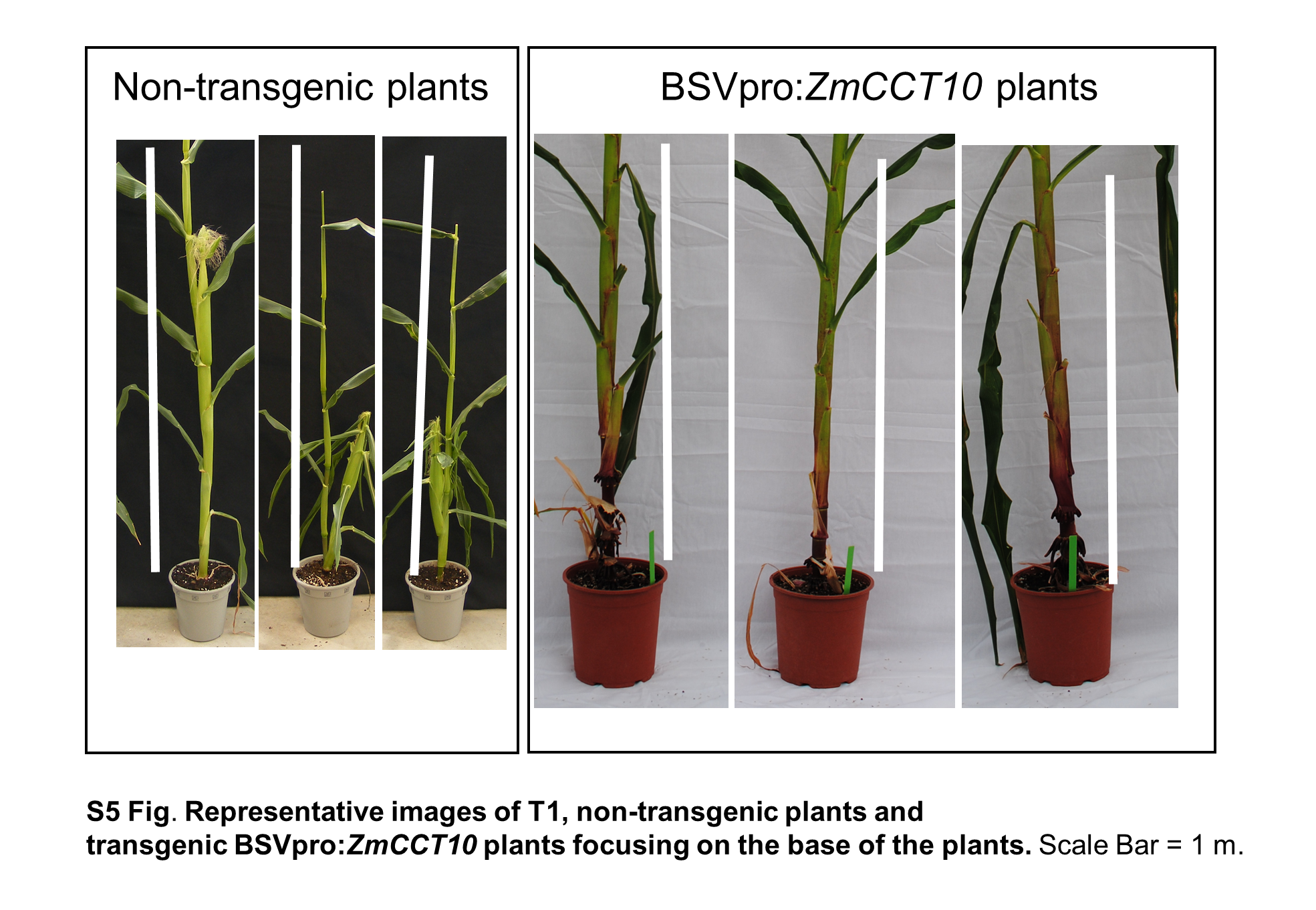

Supplement: S5 Fig — Scale Bar = 1 m. (TIF) [file pone.0203728.s005.tif]

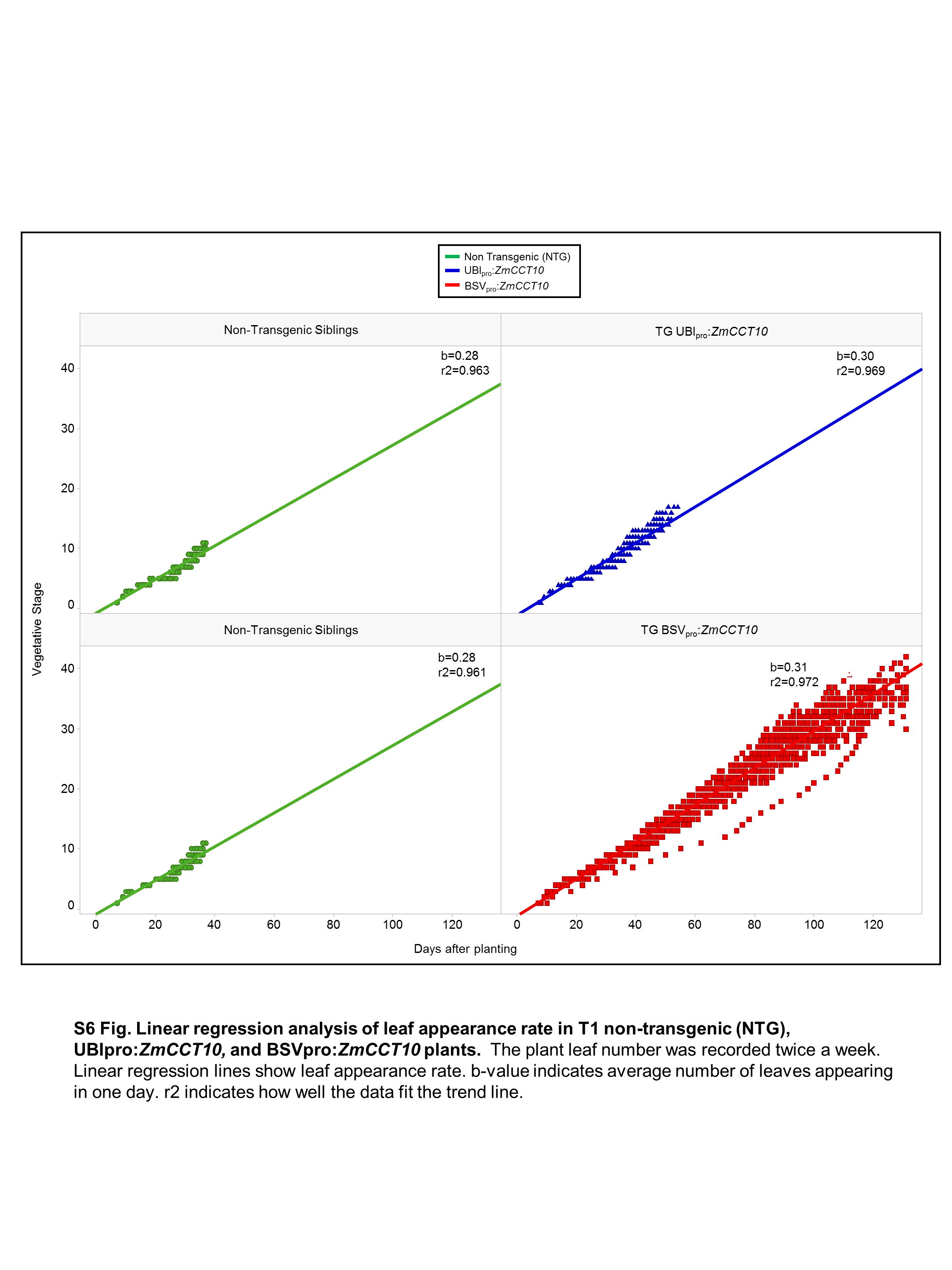

Supplement: S6 Fig — The plant leaf number was recorded twice a week. Linear regression lines show leaf appearance rate. b-value indicates average number of leaves appearing in one day. r2 indicates how well the data fit the trend line. (TIF) [file pone.0203728.s006.TIF]

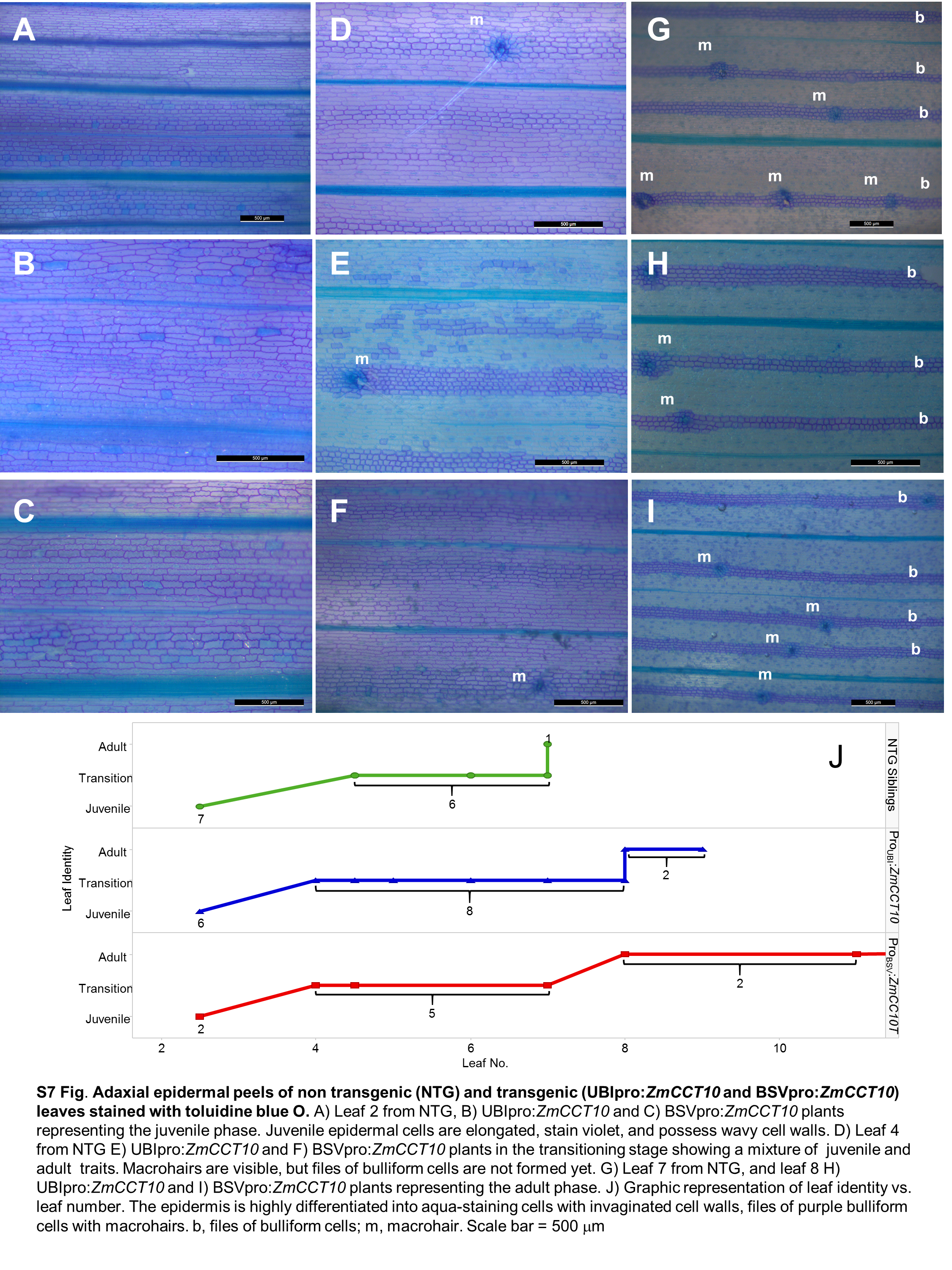

Supplement: S7 Fig — A) Leaf 2 from NTG, B) UBIpro:ZmCCT10 and C) BSVpro:ZmCCT10 plants representing the juvenile phase. Juvenile epidermal cells are elongated, stain violet, and possess wavy cell walls. D) Leaf 4 from NTG E) UBIpro:ZmCCT10 and F) BSVpro:ZmCCT10 plants in the transitioning stage showing a mixture of juvenile and adult traits. Macrohairs are visible, but files of bulliform cells are not formed yet. G) Leaf 7 from NTG, and leaf 8 H) UBIpro:ZmCCT10 and I) BSVpro:ZmCCT10 plants representing the adult phase. J) Graphic representation of leaf identity vs. leaf number. The epidermis is highly differentiated into aqua-staining cells with invaginated cell walls, files of purple bulliform cells with macrohairs. b, files of bulliform cells; m, macrohair. Scale bar = 500 mm. (TIF) [file pone.0203728.s007.tif]

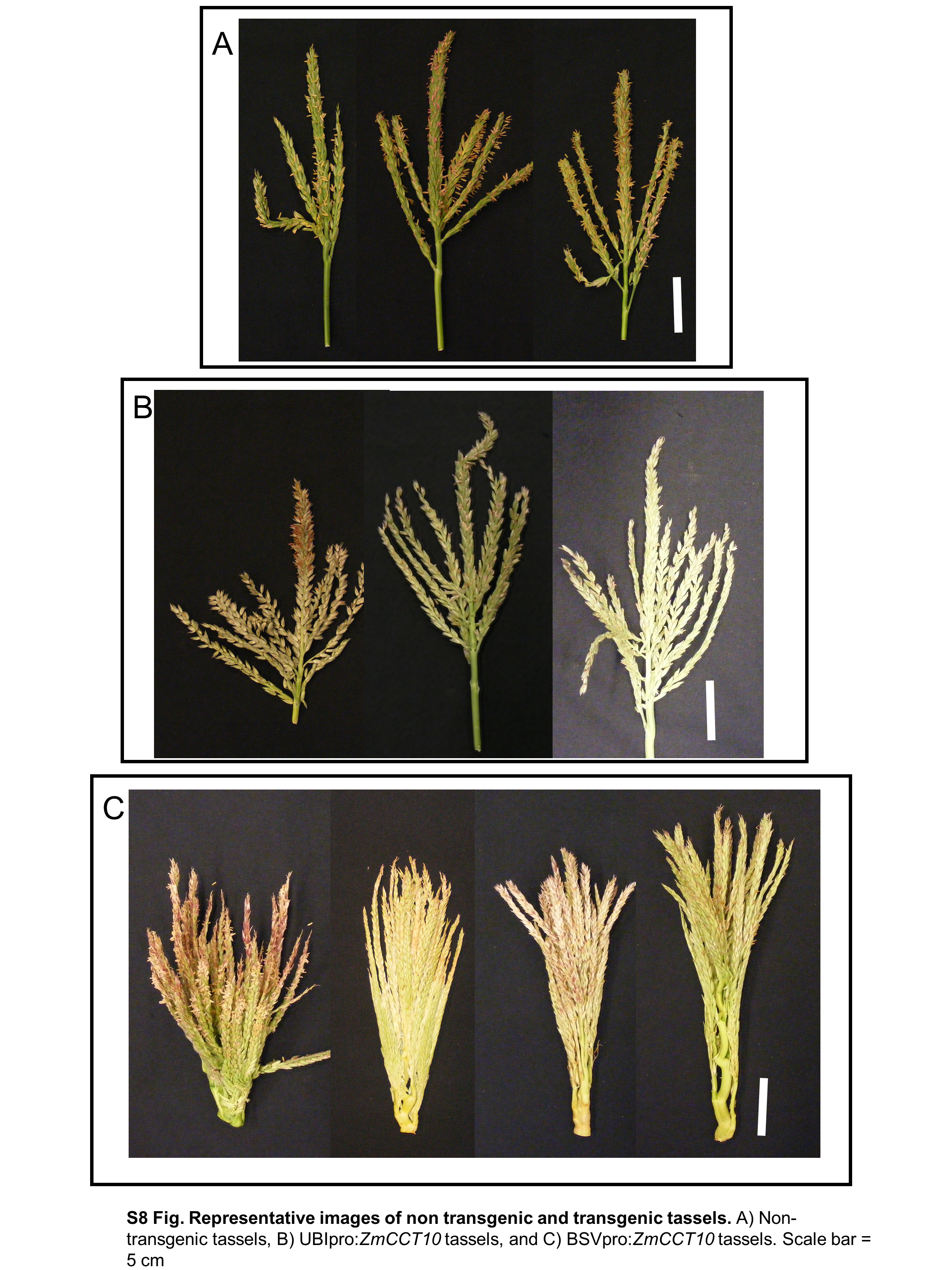

Supplement: S8 Fig — A) Non-transgenic tassels, B) UBIpro:ZmCCT10 tassels, and C) BSVpro:ZmCCT10 tassels. Scale bar = 5 cm (TIF) [file pone.0203728.s008.tif]

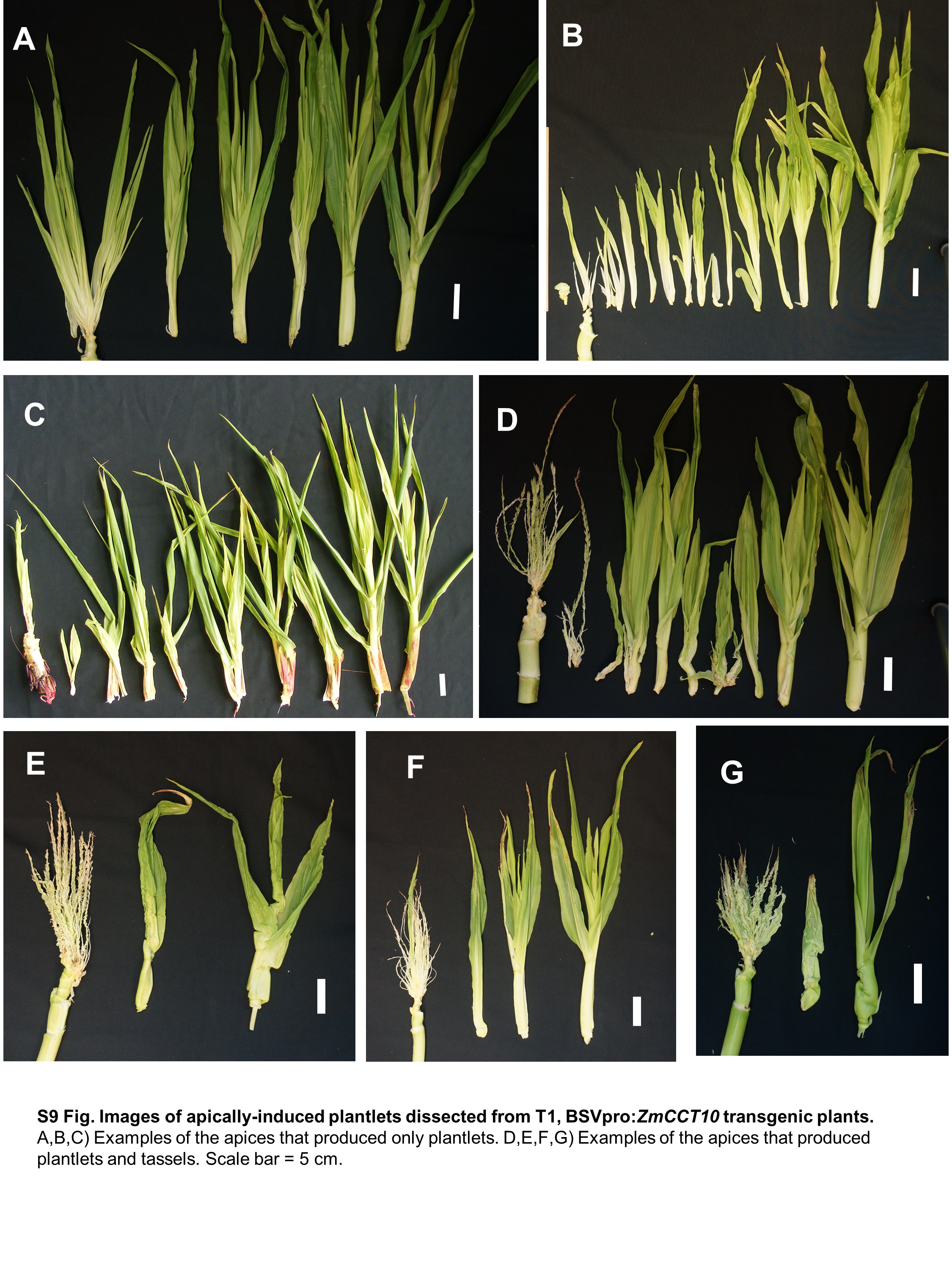

Supplement: S9 Fig — A,B,C) Examples of the apices that produced only plantlets. D,E,F,G) Examples of the apices that produced plantlets and tassels. Scale bar = 5 cm. (TIF) [file pone.0203728.s009.tif]

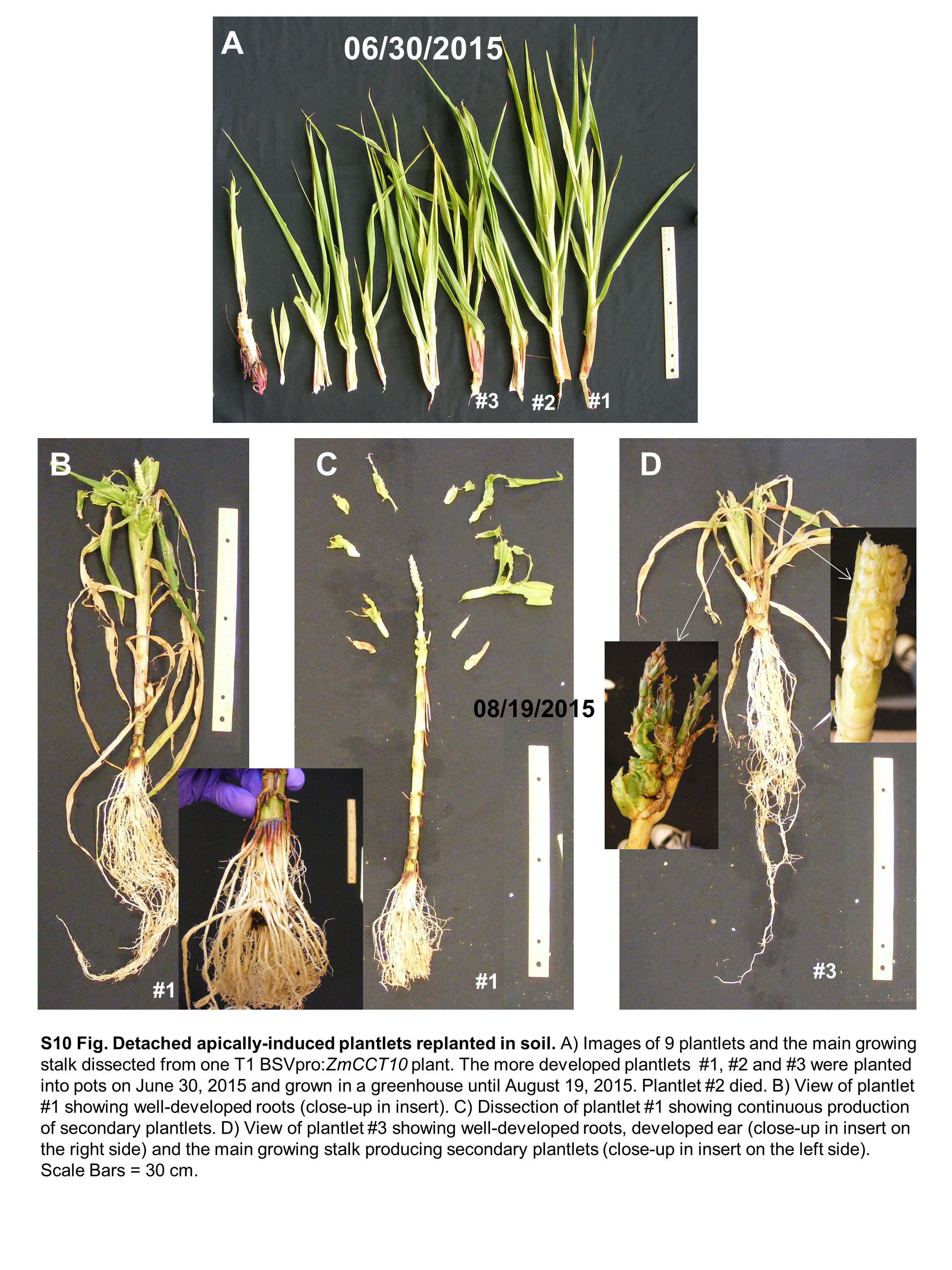

Supplement: S10 Fig — A) Images of 9 plantlets and the main growing stalk dissected from one T1 BSVpro:ZmCCT10 plant. The more developed plantlets #1, #2 and #3 were planted into pots on June 30, 2015 and grown in a greenhouse until August 19, 2015. Plantlet #2 died. B) View of plantlet #1 showing well-developed roots (close-up in insert). C) Dissection of plantlet #1 showing continuous production of secondary plantlets. D) View of plantlet #3 showing well-developed roots, developed ear (close-up in insert on the right side) and the main growing stalk producing secondary plantlets (close-up in insert on the left side). Scale Bars = 30 cm. (TIF) [file pone.0203728.s010.TIF]

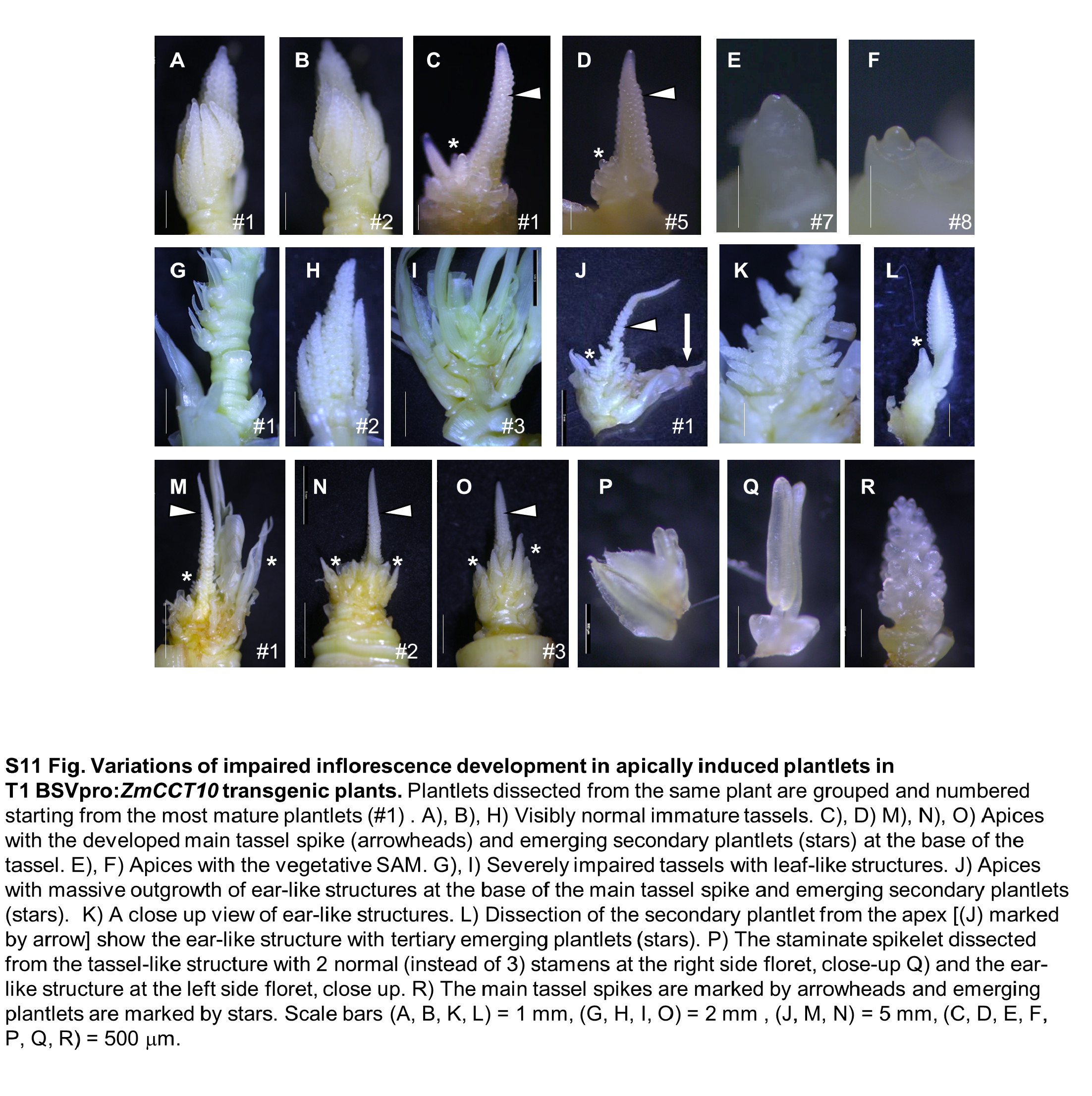

Supplement: S11 Fig — Plantlets dissected from the same plant are grouped and numbered starting from the most mature plantlets (#1). A), B), H) Visibly normal immature tassels. C), D) M), N), O) Apices with the developed main tassel spike (arrowheads) and emerging secondary plantlets (stars) at the base of the tassel. E), F) Apices with the vegetative SAM. G), I) Severely impaired tassels with leaf-like structures. J) Apices with massive outgrowth of ear-like structures at the base of the main tassel spike and emerging secondary plantlets (stars). K) A close-up view of ear-like structures. L) Dissection of the secondary plantlet from the apex [(J) marked by arrow] show the ear-like structure with tertiary emerging plantlets (stars). P) The staminate spikelet dissected from the tassel-like structure with 2 normal (instead of 3) stamens at the right-side floret, close-up Q) and the ear-like structure at the left side floret, closeup. R) The main tassel spikes are marked by arrowheads and emerging plantlets are marked by stars. Scale bars (A, B, K, L) = 1 mm, (G, H, I, O) = 2 mm, (J, M, N) = 5 mm, (C, D, E, F, P, Q, R) = 500 mm. (TIF) [file pone.0203728.s011.tif]

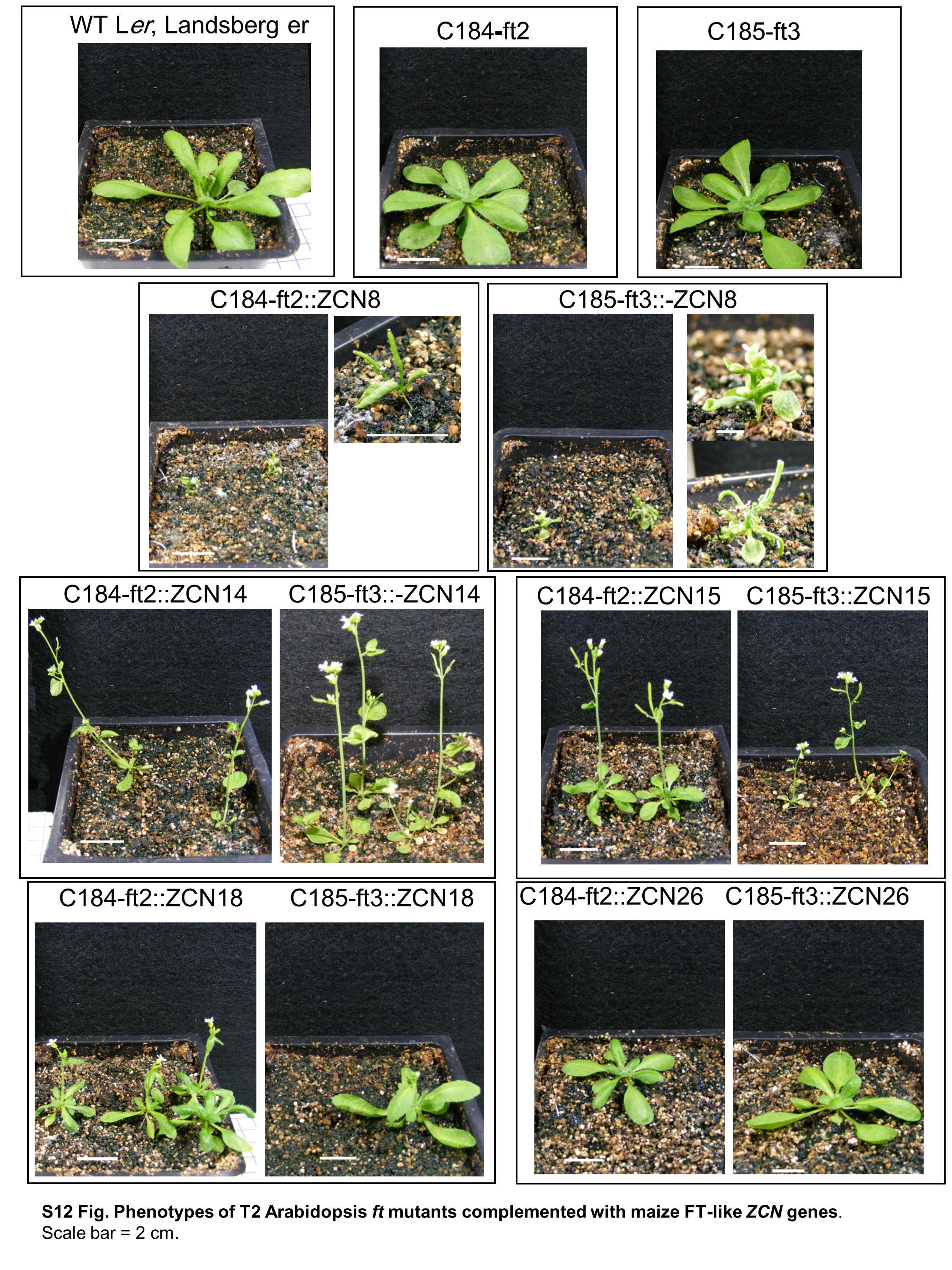

Supplement: S12 Fig — Scale bar = 2 cm. (TIF) [file pone.0203728.s012.TIF]

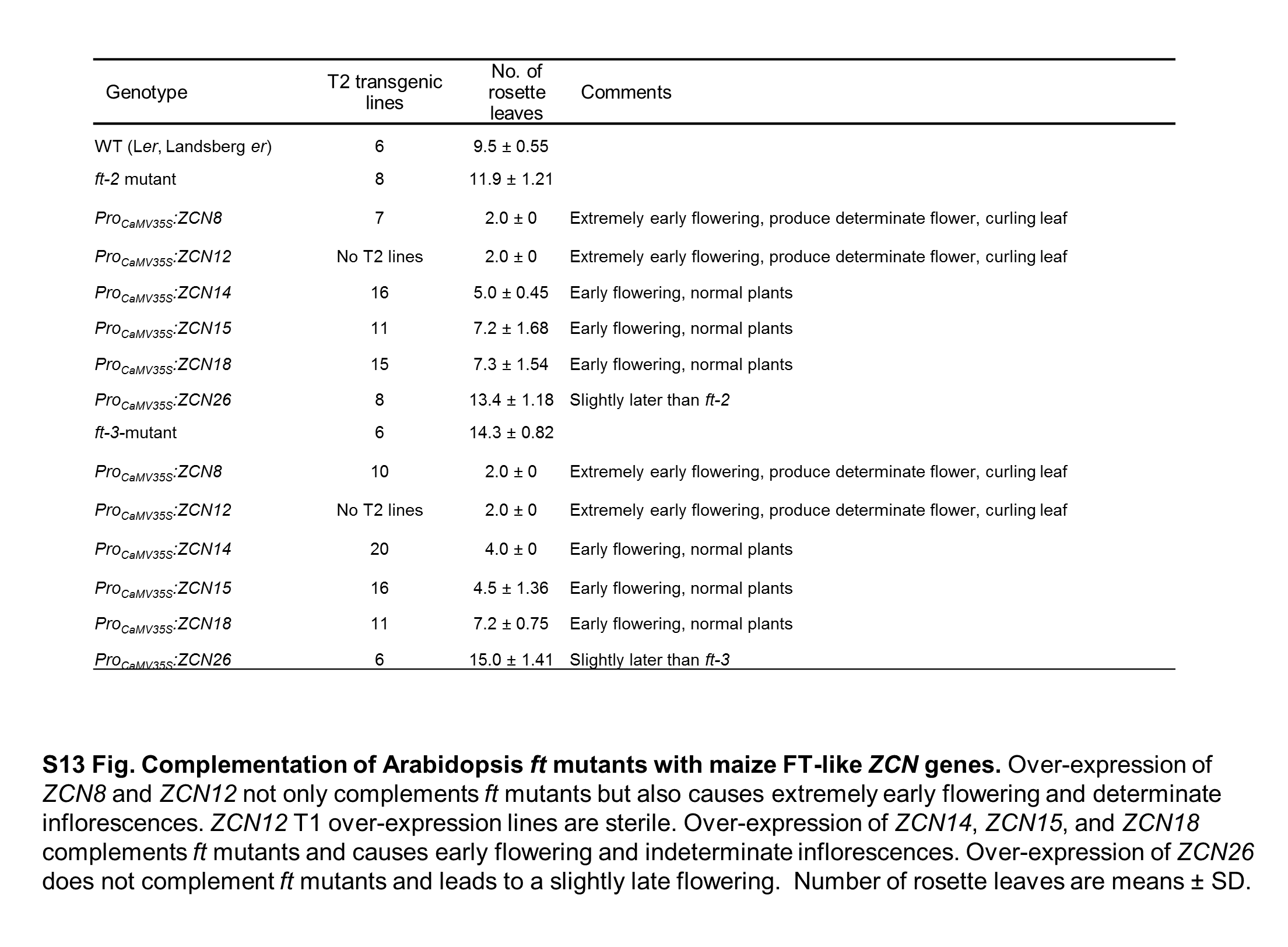

Supplement: S13 Fig — Over-expression of ZCN8 and ZCN12 not only complements ft mutants but also causes extremely early flowering and determinate inflorescences. ZCN12 T1 over-expression lines are sterile. Over-expression of ZCN14, ZCN15, and ZCN18 complements ft mutants and causes early flowering and indeterminate inflorescences. Over-expression of ZCN26 does not complement ft mutants and leads to a slightly late flowering. Number of rosette leaves are means ± SD. (TIF) [file pone.0203728.s013.tif]

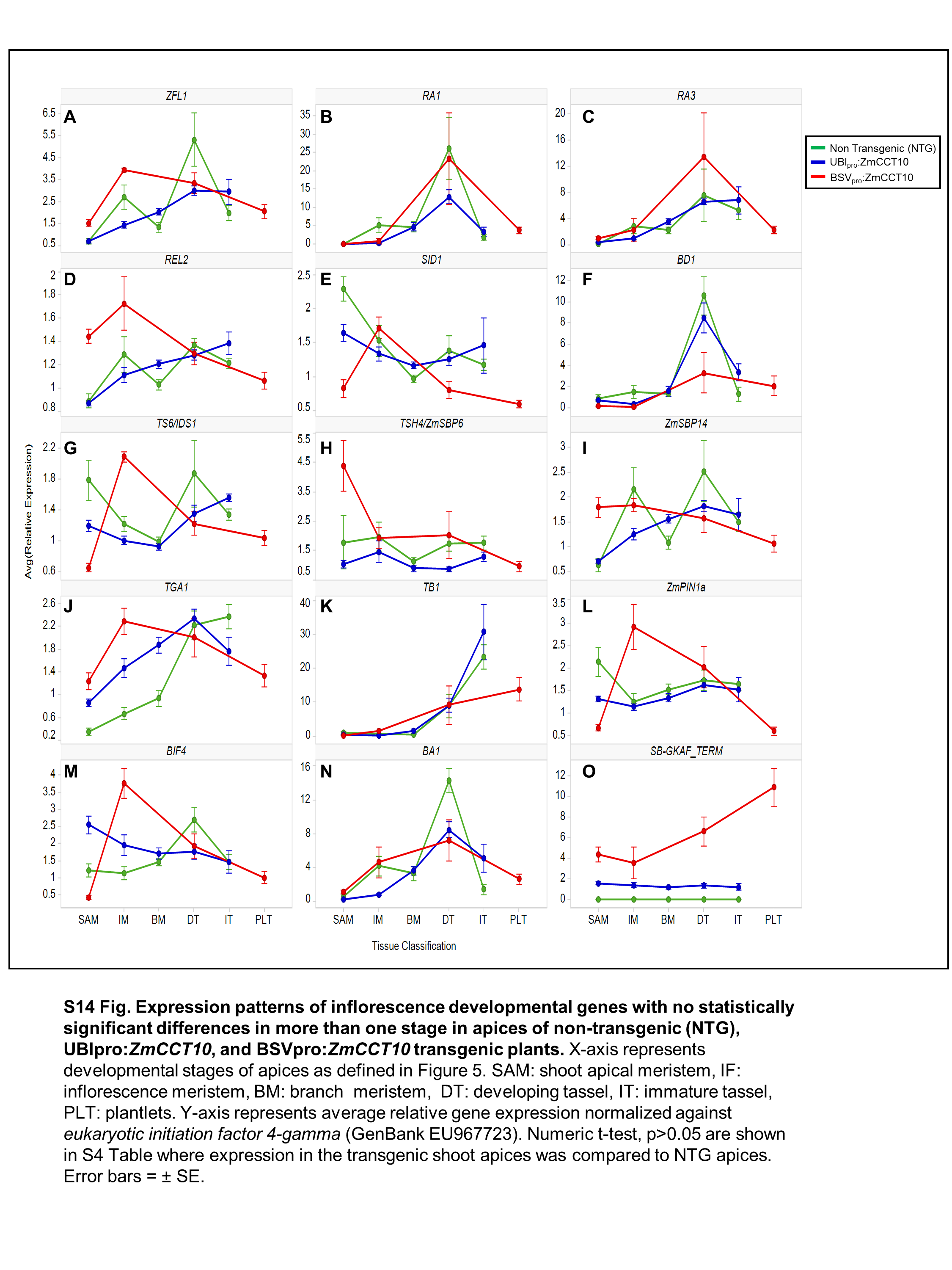

Supplement: S14 Fig — X-axis represents developmental stages of apices as defined in Fig 5. SAM: shoot apical meristem, IF: inflorescence meristem, BM: branch meristem, DT: developing tassel, IT: immature tassel, PLT: plantlets. Y-axis represents average relative gene expression normalized against eukaryotic initiation factor 4-gamma (GenBank EU967723). Numeric t-test, p>0.05 are shown in S4 Table where expression in the transgenic shoot apices was compared to NTG apices. Error bars = ± SE. (TIF) [file pone.0203728.s014.TIF]

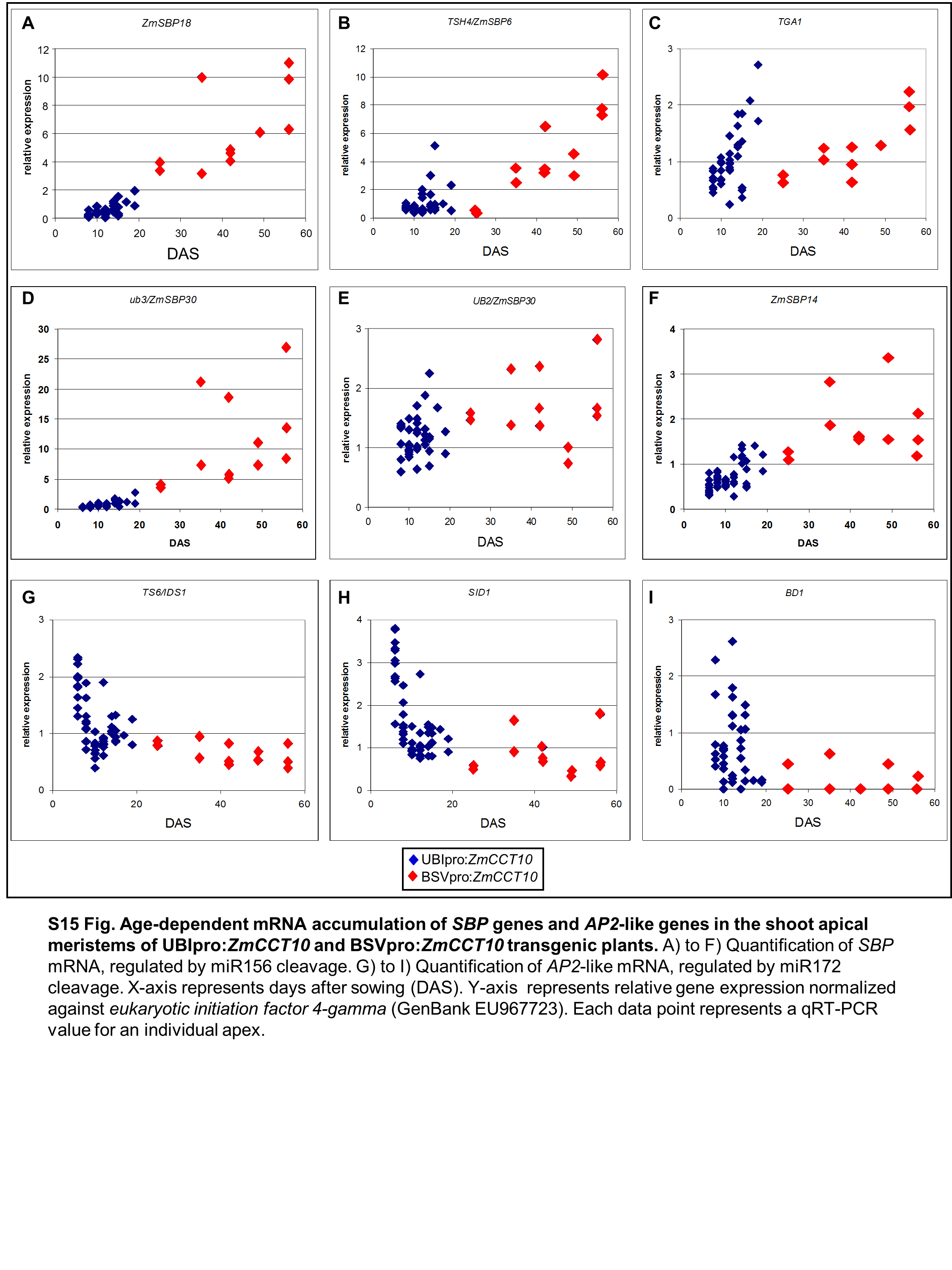

Supplement: S15 Fig — A) to F) Quantification of SBP mRNA, regulated by miR156 cleavage. G) to I) Quantification of AP2-like mRNA, regulated by miR172 cleavage. X-axis represents days after sowing (DAS). Y-axis represents relative gene expression normalized against eukaryotic initiation factor 4-gamma (GenBank EU967723). Each data point represents a qRT-PCR value for an individual apex. (TIF) [file pone.0203728.s015.TIF]
